# Using conversation analysis to explore feedback on resident performance

**DOI:** 10.1007/s10459-019-09887-4

**Published:** 2019-04-02

**Authors:** Marrigje E. Duitsman, Marije van Braak, Wyke Stommel, Marianne ten Kate-Booij, Jacqueline de Graaf, Cornelia R. M. G. Fluit, Debbie A. D. C. Jaarsma

**Affiliations:** 1 Department of Internal Medicine, Radboudumc Health Academy, Radboud University Medical Center, Gerard van Swietenlaan 4, P.O. Box 9101, 6500 HB Nijmegen, The Netherlands; 2 Department of General Practice Training, Erasmus Medical Center, Rotterdam, The Netherlands; 3 Center for Language Studies, Radboud University, Nijmegen, The Netherlands; 4 Department Gynaecologic Oncology, Erasmus Medical Center, Rotterdam, The Netherlands; 5 Department of Internal Medicine, Radboud University Medical Center, Nijmegen, The Netherlands; 6 Department for Research in Learning and Education, Radboudumc Health Academy, Radboud University Medical Center, Nijmegen, The Netherlands; 7 Centre for Education Development and Research in Health Professions, University Medical Center Groningen, Groningen, The Netherlands

**Keywords:** Postgraduate medical education, Feedback on resident’s performance, Conversation analysis, Program director, Resident

## Abstract

Feedback on clinical performance of residents is seen as a fundamental element in postgraduate medical education. Although literature on feedback in medical education is abundant, many supervisors struggle with providing this feedback and residents experience feedback as insufficiently constructive. With a detailed analysis of real-world feedback conversations, this study aims to contribute to the current literature by deepening the understanding of how feedback on residents’ performance is provided, and to formulate recommendations for improvement of feedback practice. Eight evaluation meetings between program directors and residents were recorded in 2015–2016. These meetings were analyzed using conversation analysis. This is an ethno-methodological approach that uses a data-driven, iterative procedure to uncover interactional patterns that structure naturally occurring, spoken interaction. Feedback in our data took two forms: feedback as a unidirectional activity and feedback as a dialogic activity. The unidirectional feedback activities prevailed over the dialogic activities. The two different formats elicit different types of resident responses and have different implications for the progress of the interaction. Both feedback formats concerned positive as well as negative feedback and both were often mitigated by the participants. Unidirectional feedback and mitigating or downplaying feedback is at odds with the aim of feedback in medical education. Dialogic feedback avoids the pitfall of a program director-dominated conversation and gives residents the opportunity to take ownership of their strengths and weaknesses, which increases chances to change resident behavior. On the basis of linguistic analysis of our real-life data we suggest implications for feedback conversations.

## Introduction

In the context of training residents to become medical specialists, feedback on clinical performance is fundamental to enhance their learning and confirm, restructure, tune, expand and overwrite their clinical performance (Hattie and Timperley 2007; Archer 2010; Ridder et al. 2015; Iobst et al. 2010). Literature on feedback in medical education is abundant with recommendations for giving effective feedback (Lefroy et al. 2015; Cantillon and Sargeant 2008; Sargeant and Mann 2010; Ramani and Krackov 2012; Bienstock et al. 2007; Eichna 1983; Archer 2010; Kornegay et al. 2017). In medical practice, however, providing effective feedback still seems difficult (Dudek et al. 2005; Kluger and DeNisi 1996; Archer 2010; Bing-You and Trowbridge 2009) and residents continue to be unsatisfied with the feedback they receive (Al-Mously et al. 2014; De et al. 2004; Sender Liberman et al. 2005). Apparently, there is an undesirable gap between feedback theory and practice when it comes to resident training.

A reason for this undesirable mismatch between theory and practice could be that, despite the large amount of literature on feedback in medical education, recommendations for effective feedback are scarcely supported by analyses of real-world feedback conversations. We aim to contribute to current literature by deepening the understanding of how feedback on residents’ performance is provided, and formulating recommendations for improving feedback practice. A summary of current insights from the literature on effective feedback is given below.

Effective feedback requires consideration of both feedback content and the feedback process (Cantillon and Sargeant 2008). The effectiveness of feedback increases when it is given frequently, positively framed, encouraging, accompanied by explanation to increase understanding, does not threaten the receiver’s self-esteem, recognizes the recipient’s perspectives and self-assessments, and focuses on setting goals for the future and developing an action plan (Ridder et al. 2015; Van de Ridder et al. 2015; Bing-You et al. 2017, 2018; Ramani et al. 2018; Boehler et al. 2006; Hattie and Timperley 2007).

Furthermore, verbally provided feedback should ideally be a ‘conversation about performance’ rather than a one-way transmission of information (Sargeant et al. 2009; Cantillon and Sargeant 2008; Archer 2010; Bing-You et al. 2018; Fluit et al. 2013; Boud and Molloy 2013). Passively received feedback does not lead to performance improvement, because receivers need the opportunity to analyze the feedback, ask questions about it and connect it with prior understandings to change their behavior and performance (Nicol 2010; Yang and Carless 2013).

Besides, several studies have investigated audio or video recordings of feedback conversations between clinical supervisors and medical students (Blatt et al. 2008; Ferguson 2010; Hasley and Arnold 2009; Holmboe et al. 2004; Spanager et al. 2015). They found that the conversations tended to be teacher-dominated and mostly contained general, positive statements about student behavior. A study on verbal feedback in mini clinical evaluation exercises found that, in order to be effective, feedback needs to be interactive so trainees can take ownership of their strengths and weaknesses (Holmboe et al. 2004). Despite this body of knowledge on effective feedback, in residency training both feedback givers and receivers are not satisfied.

To be able to provide meaningful additions to current feedback theories and improve feedback practice in resident training we need to take a closer look into real-world feedback practice. This approach shows promising results in the context of secondary education, where analysis of feedback in real-life teacher–student encounters yielded several practice-derived recommendations for teachers (Skovholt 2018). To our best knowledge, there are no reports of such a detailed analysis of feedback situations in the context of resident training.

To shed more light on how feedback is constructed in real-world practice and to provide practical advice on how effective feedback dialogues are created in the real-world, we chose to examine the interactional process in detail as it evolves from moment to moment by performing a fine-grained analysis of feedback conversations between program directors and residents.

## Methods

### Background

In the Netherlands, the program director (PD) is responsible for the residency program and assessment of residents’ performance. Residents must meet with their PD at least semi-annually. The purpose of these meetings is to provide feedback on residents’ overall clinical performance and set goals to bridge the gap between current and desired performance. All PDs receive a 2-day training on how to supervise and assess residents, in which they also practice providing feedback that meets the latest recommendations from the feedback literature.

### Participants and ethical approval

Participants were invited by email. To ensure heterogeneity in the sample, we recruited participants from different medical disciplines, hospitals, years of PD experience and years of resident training (Table 1). Original data were treated confidentially and analyses were performed anonymously. All participants provided written informed consent. The study was approved by the Netherlands Association for Medical Education (file number 566).

**Table 1:** Participants

	Medical specialty	Hospital (general hospital/university medical center)	Program Director male/female years of experience as PD	Resident male/female year of training	Duration of meeting minutes
1	Internal medicine	General hospital	Male 10 years	Male 3rd year	33:19
2	Radiology	University medical center	Female 3 years	Male 2nd year	40:01
3	Radiology	University medical center	Male 1 year	Male 1st year	41:21
4	Internal Medicine	General hospital	Female 2 years	Female 3rd year	30:05
5	Internal Medicine	University medical center	Female 1 year	Female 2nd year	28:28
6	Radiology	General hospital	Female 5 years	Male 3rd year	23:52
7	Surgery	General hospital	Male 8 years	Female 1st year	12:32
8	Surgery	University medical center	Male 5 years	Male 4th year	53:53

### Analytic procedure

We used Conversation Analysis (CA) to analyze the data of the meetings. CA is an ethnomethodological approach to interaction aimed at uncovering interactional patterns that structure naturally occurring, spoken interaction (Mazeland 2006; Maynard and Heritage 2005; Sidnell and Stivers 2012). It is a data-driven and iterative procedure. CA provides tools for detailed, practice-based analysis of interaction and has proved to be very valuable in (medical) educational research (Sidnell and Stivers 2012). Therefore, this methodology is particularly suited to the aim of our research.

The recorded interactions were transcribed using Jeffersonian transcription conventions, in which many symbols are used to transcribe what has been said and noting how it has been said (Jefferson 1986) (see “Appendix 1”). Since CA is an inductive qualitative linguistic method, it seeks to describe and explain the structures of social interaction “through a reliance on case-by-case analysis leading to generalizations across cases but without allowing them to congeal into an aggregate” (Sidnell and Stivers 2012, p. 2). Our analysis, therefore, started with an explorative stage of making observations of details of the interaction in individual fragments. This led to working hypotheses on aspects of the interaction (e.g., how actions, like challenging an opinion or closing the interaction, are performed), which were then checked against the entire dataset. By going back and forth between various individual fragments, we developed a description of the interaction that was grounded in individual fragments and simultaneously applicable to the entire data set (Sidnell and Stivers 2012). Accordingly, the analyses of extracts presented in the results section are illustrative of thorough microanalyses of feedback sequences throughout the entire dataset.

Two researchers (MD and MB) identified 62 fragments in which resident performance was discussed in the recordings of our study. The rest of the conversations concerned practical issues like planning, logistics and niceties. Two data sessions with a group of six experienced CA researchers from all over the country were held in which specific fragments were analyzed, a procedure typical for CA methodology (Ten Have 2007). After these two data sessions, MD and MB analyzed the rest of the data and periodically reflected on their findings with WS in three separate meetings. Finally, the findings were discussed in the entire research team.

The primary researchers (MD, MB) had no direct connection with the participants. MD is a medical doctor with experience as a resident in a general teaching hospital. This study is part of her Ph.D. research in medical education. MB is an educationalist and researcher in medical education with a background in linguistics. WS is an assistant professor in language and communication, working as a researcher using Conversation Analysis in medical settings. The results were discussed in the research team (MKB, JG, CF, DJ). MKB and JG are program directors in a university medical center, CF is a medical doctor and an educationalist working as post-doctoral educational researcher with expertise on feedback in residency programs and DJ is a professor in medical education. MD and MB built an audit trail by documenting the analytical decisions and summaries of the discussions with the research team.

## Results

Eight meetings between PDs and residents were recorded in 2015–2016. They lasted between 12.32 and 53.53 min, with an average of 32.56 min, which resulted in 262.42 min of audio recording.

The analysis revealed that the two feedback formats described in the literature, a one-way transmission of information versus a conversation about performance, consistently appeared throughout the data set. In our study, we defined these types of feedback as unidirectional and dialogic feedback activities. Unidirectional feedback activities are characterized by an evaluation on past performance posted by the program director. Dialogic activities are characterized by an initial question, followed by in-depth questions of the program director to ask the resident about past performance.

Feedback as unidirectional activity occurred in 41 and feedback as a dialogic activity in 21 of the 62 fragments. Below, we first present examples of unidirectional feedback (positive and negative feedback examples) and then two examples of feedback as a dialogic activity. To present the transcribed data we use a two-line lay-out with the original Dutch language first and a literal English translation line next. The focus of our analysis is on how the two feedback formats are interactionally constructed by the PD and resident. As is common in CA research, we include CA literature in this section to validate the analyses of the presented fragments (Veen and de la Croix 2016; Sidnell and Stivers 2012).

### Unidirectional feedback activities

Unidirectional feedback activities have three interactional features: (1) the PD introduces an aspect of performance for feedback, (2) this aspect is then evaluated by the PD, and (3) the resident’s responses are minimal.

### Positive evaluation of performance

A typical example of a positive performance evaluation is presented in Extract 1. The feedback sequence is initiated by the PD after a short silence following the closing of the preceding topic.
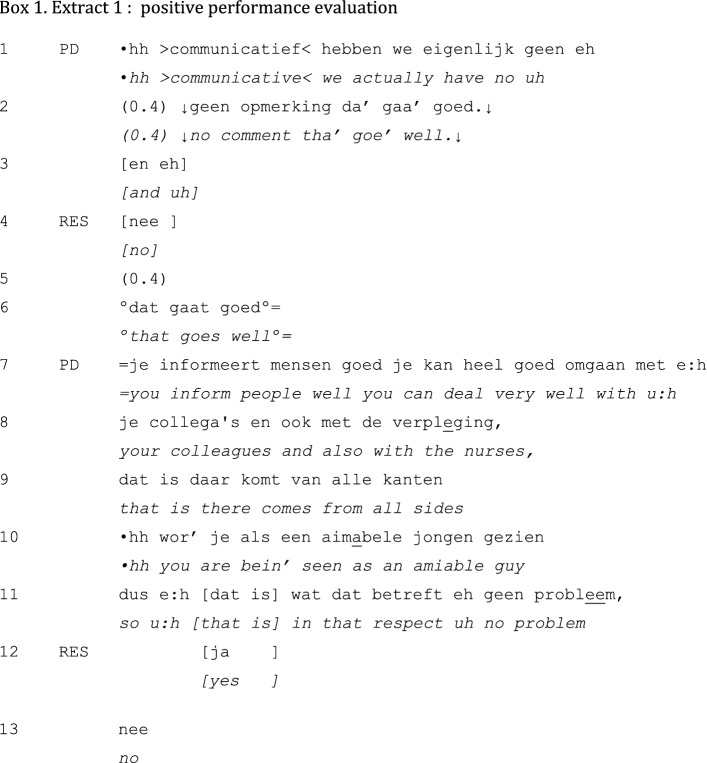


The PD initiates this sequence by mentioning its topic: “communication” (line 1). Before providing his evaluation, “that goes well” (line 2), he states that there was no comment on that aspect of performance (lines 1–2)—most likely from supervisors of the resident. In overlap with a possible addition from the PD to the evaluation (line 3), the resident responds with a minimal “no” (line 4)—probably in response to the turn part “no comment”—and after a brief silence (line 5) the resident continues with a softly spoken repetition of the PD’s evaluation “that goes well” (line 6). Once the resident has demonstrated his reception of the evaluation, the PD proceeds with providing several pieces of detailed evidence that support his initial evaluation (lines 7–10). Only upon having started uttering the conclusion, launched with a conclusive “so” (line 11), the resident confirms the PD’s previous contribution (line 12). The conclusion “so eh in that respect that is eh no problem” is again responded to by a minimal “no” (line 13), upon which the PD launches the next feedback sequence (not shown).

Resembling Extract 1, positive performance evaluations are generally done in brief units of discourse. They are invariably initiated by the PD; the resident’s contribution to the interaction is limited. The performance evaluation itself remains rather superficial (e.g., “it goes well”), although it is sometimes supported by more specific examples of good conduct. In some positive unidirectional feedback sequences, the evaluation is slightly attenuated after it has been posed. In Extract 2, for example, the resident downplays the PD’s positive evaluation of the competency ‘Scholar’:
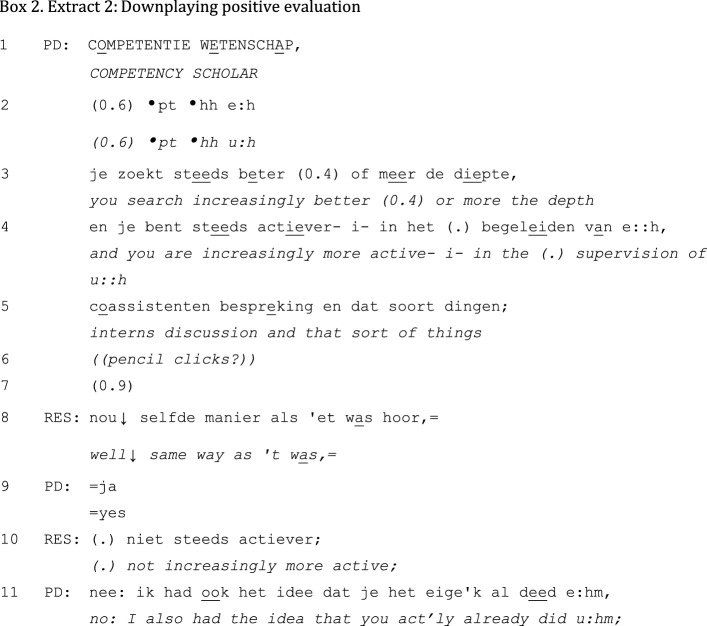


After a short gap following the PD’s positive evaluation “you are increasingly more active- i- in the (.) supervision of u::h interns discussion and that sort of things” (lines 4–5), the resident downplays the evaluation by saying that he thinks that it is still the same as it was before. This downplaying is accepted by the PD with a minimal “yes” in line 9, which is elaborated in line 11.

### Negative evaluation of performance

The interactional structure of negative evaluations in unidirectional feedback activities differs from that of positive evaluation interactions. An example of a negative performance evaluation is presented in Extract 3. In this extract, the PD introduces the topic by stating that there have been strong critiques as well as positive comments on the resident’s performance in general:
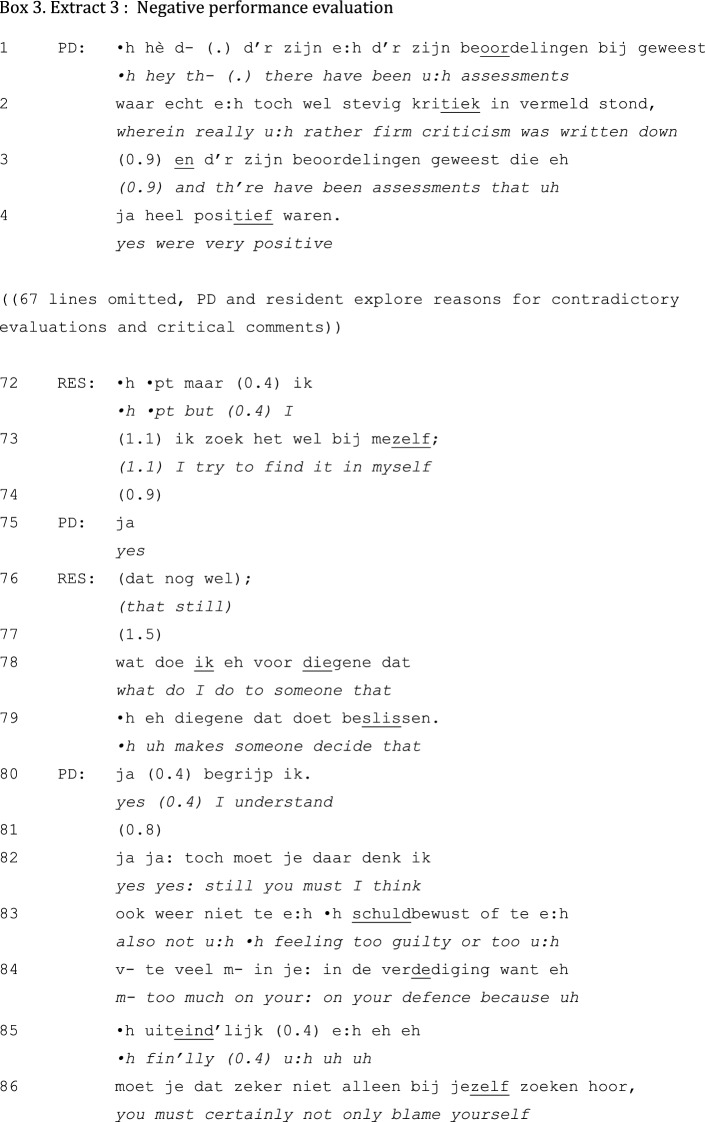

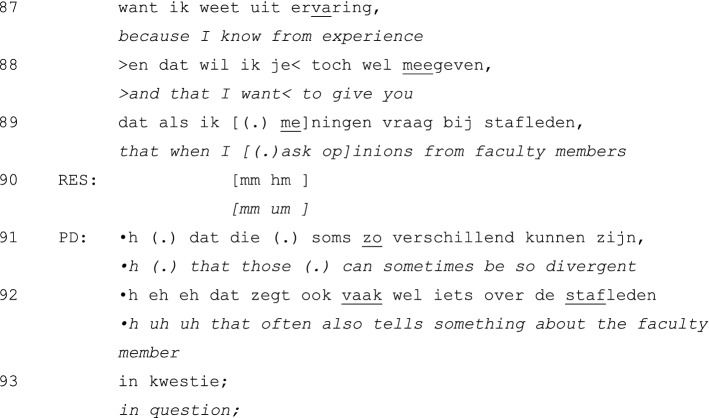
 After the problem-initiation sequence, in which the PD topicalizes the rather strong criticisms of faculty members (lines 1–2), he comments on other feedback that has been very positive (lines 3–4). The latter might be understood as mitigating negative feedback, but in this extract it seems to initiate a collaborative exploration of potential causes for the divergent feedback (not presented). This discussion culminates in a hesitantly produced conclusion by the resident that he blames his own behavior (lines 72–73). This self-deprecating evaluation (Pomerantz and Heritage 2012) gets a minimal response from the PD (line 75) following a short silence (line 74), after which the resident pursues his turn (line 76). This time, the resident does not wait for the PD to respond, but again resumes the talk once the PD’s silence emerges.

Following Pomerantz’ analysis (Pomerantz and Heritage 2012), the resident might interpret the absence of an overt disagreement with the self-deprecation in lines 74–75 and 77 as an implicit confirmation of the self-deprecation. As agreement with self-deprecation is not preferred (Pomerantz and Heritage 2012), the unfolding of this sequence so far is potentially problematic. In the following turns, however, the sequence gets a more favorable continuation: after claiming to have understood (line 80), the PD proceeds with an explicit statement of disagreement (lines 82–86). Without completely freeing the resident of his ‘guilt’ (line 83), the PD reduces the ‘burden’ by pointing at faculty members as part of the problem. He accounts for this relocation of responsibility by referring to his experience with these issues (line 87). In the following interaction (not shown), the PD slightly elaborates his account—resident turns being limited to minimal responses and backchanneling (as in line 90)—and ends the interaction with an advice-giving sequence.

This excerpt is exemplary of the general tendency of PDs to mitigate negative other-evaluations and downplay critical self-evaluations. Both practices seem to result from an orientation toward negative assessments as socially problematic activities (Skovholt 2018; Asmuß 2008).

### Dialogic feedback activities

In contrast to unidirectional feedback fragments, dialogic feedback fragments are more interactive. These fragments are initiated by the PD asking a question to invite the resident to introduce a topic for discussion. The PD leaves room for the resident’s narration and elicits further elaboration on the topic. An example of dialogic feedback in a conversation between two PDs and one resident is presented in Extract 4.
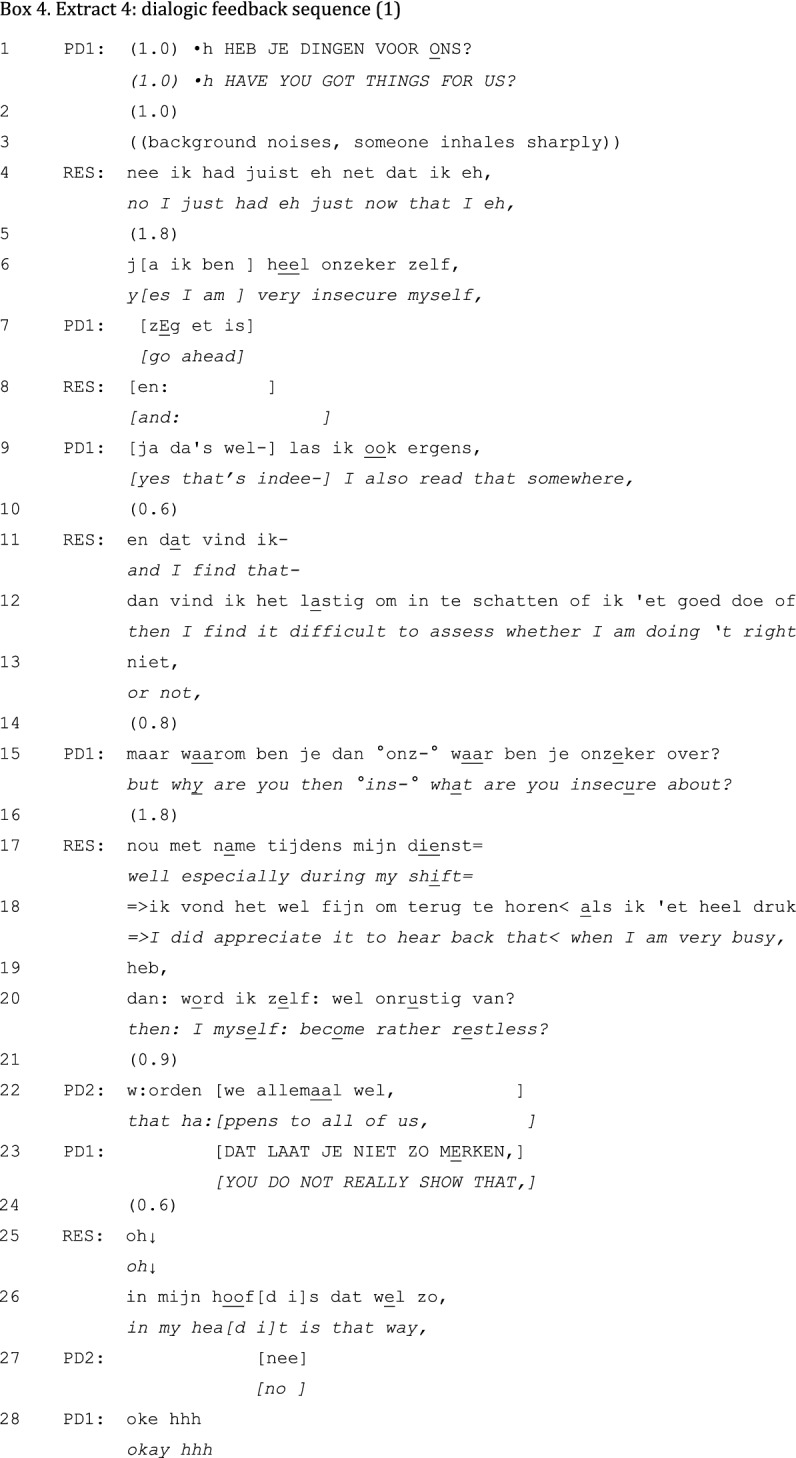


The interaction in this fragment occurs at the end of a range of feedback sequences. The PD’s question “Do you have things for us?” opens up the floor to yet unmentioned topics (Schegloff and Sacks 1973). The resident’s response is delayed and hesitant (lines 2–7). In partial overlap with the PD’s “go ahead” (line 7), the resident proposes an issue for discussion (“yes I am very insecure myself”, line 6) and elaborates on that in lines 11–13. In the following interaction both PDs challenge the validity of the problem, first directly (line 15) and then with an outright invalidation of the problem: “that happens to all of us” (line 22). PD1’s response in line 23 contains another mitigation of the issue: “you don’t show it”. These challenges appear interactionally problematic: they are followed by a silence (line 24), received with “oh” (line 25), and objected with “in my head it is that way”. Despite PD1’s closure-implicative “okay” (Stokoe and Edwards 2008), the resident again takes the turn to explain his issue—which gets mitigated, is raised anew, and gets mitigated once more before one of the PDs formulates an advice and concludes the sequence with “that insecurity will decrease by itself” (not shown). If taking the floor is followed by repeated mitigations by the PD(s), then the result is a pseudo-dialogue (Skovholt 2018). Although the PDs’ questions “made the conversation appear ‘dialogic’” (Skovholt 2018) (p152), their disagreements with the topicalized problem quickly subverted the dialogue into a conversation in which the PDs gradually took over the agenda.

Unlike Extract 4, Extract 5 is a dialogic feedback sequence that does not incorporate mitigations (in this case, of a positive situation) and *remains* dialogic. The feedback sequence in Extract 5 has been initiated by the PD’s question “What is going well, according to you?” The resident has proposed a specific topic for discussion (“performing surgery”) and has explained why he thinks it goes well. Note that the open character of the PD’s question is fundamentally different from the assertive topicalizations and evaluations with which unidirectional feedback is started off. The consequences of this become clear as the interaction unfolds:
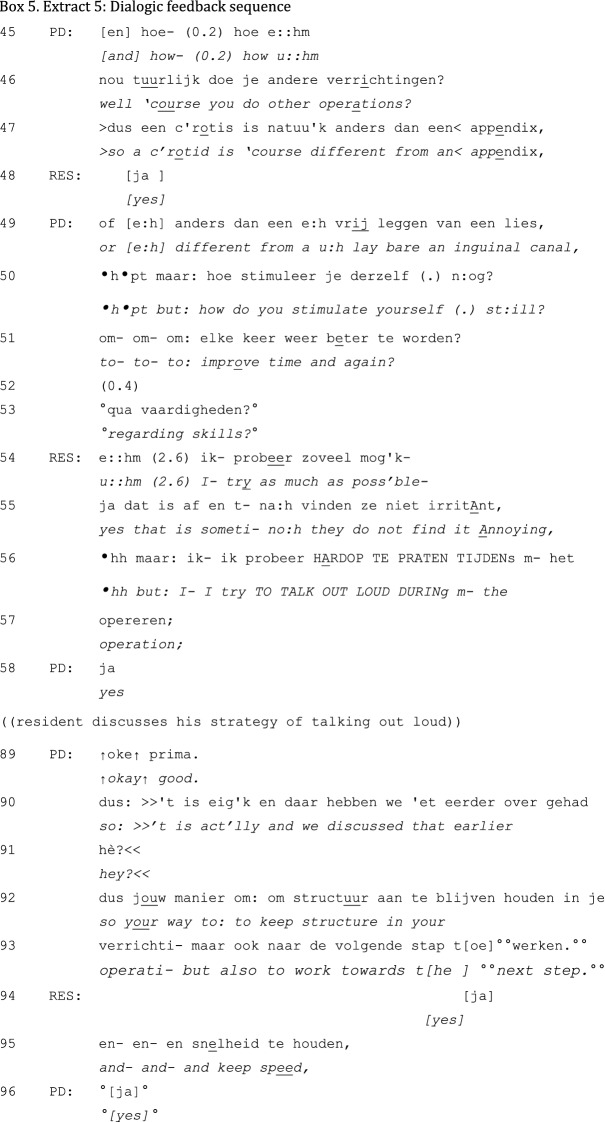


The PD’s next question is about self-development (lines 50–51), which is prefaced by and builds on the resident’s explanation. Again, this question elicits an elaborate response from the resident with minimal PD contributions (lines 59–88). The sequence is closed by a PD-initiated summary.

## Discussion

In this study, we used conversation analysis to explore how feedback is constructed in semi-annual evaluation meetings between residents and PDs in postgraduate medical education.

Although literature highlights the importance of feedback as an interactive dialogue and states that unidirectional feedback is not helpful and should be avoided (Pelgrim et al. 2013), we found that unidirectional feedback still prevails over dialogic feedback in real-world practice. Our fine-grained analysis shows how both feedback formats are interactionally constructed.

Unidirectional feedback is dominated by the PD, who initiates the feedback sequence by introducing a topic for discussion and then provides an evaluation on the clinical performance of the resident. The PD subsequently dominates the conversational floor by barely leaving any opportunity for the resident to respond. Dialogic feedback is also initiated by the PD, but here the PD asks a question to invite the resident to introduce a topic for discussion. Subsequently, the PD leaves room for the resident’s narration and elicits further elaboration on the topic by asking more questions.

The question-initiated interaction of dialogic feedback allows the resident to take regular turns at talk (Frank 1982). This enhances the dialogic nature of the interaction. Also, as shown in Extracts 4 and 5, providing conversational space benefits the opportunity for the resident to introduce “hither-to unmentioned mentionables” (Schegloff and Sacks 1973, p. 245). Providing this opportunity is a way of acknowledging the resident’s right “to introduce topics at certain junctures in the conversation” (Frank 1982, p. 359).

Dialogic feedback interactions provide the PD with ample information about residents’ performance, while avoiding the pitfall of a PD-dominated conversation. It gives residents the opportunity to take ownership of their strengths and weaknesses, which, in turn, increases the likelihood that they will apply the feedback in order to improve their future performance (Holmboe et al. 2004).

However, despite being granted the conversational floor, taking the floor seems difficult. This corroborates the importance of leaving the floor open by asking open questions to elicit residents’ responses and avoid the pitfall of occupying the floor that has just been opened to the resident.

Another important finding of our study is the pervasiveness of mitigation in feedback conversations. PDs and residents frequently downplay negative as well as positive feedback. This feature resembles a common linguistic strategy used in various types of communication: *hedging*. Hedging can be described as mitigating claims by introducing elements of doubt (e.g. “I think”, “probably”) or signaling a lack of commitment to what is said (e.g. “The management team said that…”) (Fraser 2010). Hedging has been reported to be prevalent in *written* narrative feedback on residents’ performance (Ginsburg et al. 2016). Ginsburg et al. (2016) argue for the functionality of hedging in assessment contexts in medical education for the benefit of harmonious relationships. Yet, hedging as a way to downplay feedback can also create confusion or misunderstanding of the message (Ginsburg et al. 2016; Bonnefon et al. 2011). As such, hedging does more harm than good (Ramani et al. 2017).

### Implications for practice

Based on our findings using a fine-grained conversation analysis, we suggest three implications for effective feedback practice. First, feedback providers should *open up the floor to residents*. They should ask questions to elicit information about the resident’s performance; pose explicit evaluations of performance after having enquired about the resident’s perception of his performance. Initial posing of performance evaluations limits residents’ conversational space and bars opportunities to build on residents’ evaluations of their own performance. Second, feedback providers should *leave the floor open to the resident* to further enhance the dialogic conversation. Again, they should ask questions to facilitate two-way dialogue. Additionally, feedback providers should explore issues brought up by the resident before dismissing them as irrelevant or not important. Third, feedback providers should *be careful in mitigating feedback.* Hedging might serve as a way to be polite and maintain relationships, but it can also distort the message.

### Strengths, limitations and future research

A strength of our study is that we studied real-life feedback conversations, so we were able to ground our recommendations in actual practice. The analysis could have been strengthened by using video-recordings instead of audio-recordings. Video-recordings allow including non-verbal interactional features in the analysis of feedback construction. However, participants were very hesitant to participate in a study using video-recordings and allow themselves to be video-recorded.

Furthermore, we included participants from different hospitals and medical specialties with different experience levels in training and teaching, which resulted in participant diversity. Although we deliberately chose to select different types of medical disciplines (surgical and non-surgical), we could have maximized heterogeneity of the sample by including a wider range of medical specialties. We stress the need for further studies of feedback conversations between residents and their supervisors to find out whether the conversational structures can be generalized to similar feedback settings and to different cultures. In future analyses it would also be interesting to analyze where in the overall structure of the meeting the feedback sequences tend to occur, how the feedback sessions start and end and how this changes the nature of the feedback.

## Conclusion

This study explores how feedback is constructed in semi-annual evaluation meetings between residents and PDs in postgraduate medical education. Unidirectional feedback prevails over dialogic feedback in real-world practice. Dialogic feedback avoids the pitfall of a program director-dominated conversation and gives residents the opportunity to take ownership of their strengths and weaknesses, which increases chances to change resident behavior. On the basis of linguistic analysis of our real-life data we suggest implications for feedback conversations: feedback providers should open up the floor to residents, they should leave the floor open to the resident to further enhance the dialogic conversation and they should be careful in mitigating feedback.

## Appendix 1

**Table:** Jeffersonian transcription conventions

Symbol	Name	Used to indicate
:	Colon (s)	Extended or stretched sound, syllable, or word
_____	Underline	Vocalic emphasis
(.)	Micropause	Brief pause of less than (0.2)
(1.2)	Timed pause	Intervals occuring within and between same or different speaker’s utterance
(())	Double Parentheses	Scenic details
()	Single parentheses	Transcriptionist doubt
.	Period	Falling vocal pitch
?	Question marks	Rising vocal pitch
↑↓	Arrows	Pitch resets; marked rising and falling shifts in intonation
° °	Degree signs	A passage of talk noticeably softer than surrounding talk
=	Equal signs	Latching of contiguous utterances, with no interval or overlap
[ ]	Brackets	Speech overlap
[[	Double brackets	Simultaneous speech orientations to prior turn
!	Exclamation Points	Animated speech tone
–	Hyphens	Halting, abrupt cut off of sound or word
> <	Less than/greater than signs	Portions of an utterance delivered at a pace noticeably quicker than surrounding talk
OKAY	CAPS	Extended or stretched sound, syllable, or word
hhh.hhh	H’s	Audible outbreaths, possibly laughter. The more h ’s, the longer the aspiration Aspirations with periods indicate audible inbreaths (e.g., .hhh). H ’s within (e.g., ye(hh)s) parentheses mark within-speech aspirations, possible laughter
pt	Lip smack	Often preceding an inbreath
hahheh hoh	Laugh syllable	Relative closed or open position of laughter
$	Smiley voice	Laughing/chuckling talk between markers
